# A school-based intervention based on self-determination theory to promote girls' physical activity: study protocol of the CReActivity cluster randomised controlled trial

**DOI:** 10.1186/s12889-019-6817-y

**Published:** 2019-05-06

**Authors:** Yolanda Demetriou, Joachim Bachner

**Affiliations:** Department of Sport and Health Sciences, Technical University of Munich, Georg-Brauchle-Ring 62, 80992 Munich, Germany

**Keywords:** Physical activity, Mechanisms, Behaviour change

## Abstract

**Background:**

Physical inactivity is deemed to be the fourth leading cause for premature death. Nevertheless, only a minority of children and adolescents in Germany fulfil the guideline of the World Health Organization of at least 60 min of moderate-to-vigorous physical activity per day. Children and adolescents with a lower socioeconomic background and especially girls are regarded as a high-risk group in terms of physical inactivity. Aim of this study is to examine how a theory-based physical education intervention programme supporting students’ autonomy, competence and relatedness affects physical activity both during these lessons and in leisure time. Based on the self-determination theory and the social cognitive theory, the extent to which autonomy, competence, relatedness, social support and self-efficacy in girls mediate the effect of the intervention programme on their physical activity will be examined. Moreover, the potential moderating role of socioeconomic status, environmental factors, teacher characteristics and BMI will be tested.

**Methods:**

CReActivity is a two-arm cluster randomised controlled trial with a follow-up period of three months after the end of the intervention programme. A total of 600 sixth grade girls in lower secondary schools in Bavaria, Germany will be proactively recruited. The intervention is carried out by the physical education teachers of the participating classes for five months. Primary outcome is the girls’ physical activity measured by accelerometers and systematic observations.

**Discussion:**

We expect to provide an intervention programme that can contribute to the increase of physical activity levels in girls and offer insights into the mechanisms of physical activity behaviour change.

**Trial registration:**

German Clinical Trials Register DRKS00015723 (date of registration: 2018/10/22 retrospectively registered).

**Electronic supplementary material:**

The online version of this article (10.1186/s12889-019-6817-y) contains supplementary material, which is available to authorized users.

## Background

Although it is widely known that physical inactivity is a cause of chronic diseases, such as obesity, type II diabetes, hypertension, cardiovascular diseases, colon and breast cancers, and thus, the fourth leading cause for premature death [1], a high percentage of people in industrialised countries still lead a sedentary lifestyle [2]. According to the Health Behaviour in School-aged Children (HBSC) study, a sedentary lifestyle is already observable at a young age, particularly in girls [3]. A study by Cooper, Goodman [4] analysing pooled accelerometer data from more than 27,000 children and adolescents (aged 3 to 18) shows that only 9% of the male and 2% of the female participants meet the World Health Organization (WHO) recommendation of daily 60 min moderate-to-vigorous physical activity. Concerning the development throughout childhood and adolescence, physical activity decreases on average by about 4% with each year of age after the age of six. Additionally, sedentary behaviour, which nowadays is considered not just as the opposite of physical activity but to have its own independent negative influence on health, is increasing [4, 5].

These findings underscore the need for widespread efforts to start promoting regular physical activity at an early age. However, in a recent systematic review of the effectiveness of school-based interventions, Demetriou and Höner [6] found that only 56.8% of the studies examining physical activity behaviour in children and adolescents achieved a significant – and, for the most part, small – increase. The majority of the reviewed studies suffered serious limitations. Thus, with these limitations of relevant previous studies kept in mind, further research is needed to initiate more promising and longer lasting improvements in physical activity. In the following, the most critical limitations in previous research are described and it is explained how these limitations are to be circumvented in this project.

### Lacking consideration of theoretical foundation and mediating constructs

Even though quite a few studies refer to a theoretical model (e.g., social cognitive theory, theory of planned behaviour, transtheoretical model, self-determination theory) to explain physical activity behaviour enhancement [7], very few of them explicitly design and evaluate their intervention based on the respective theory [6]. In line with the lacking consideration of theoretical models, only few studies have been carried out that systematically examine the mediating mechanisms of physical activity behaviour change in youth [8, 9]. Thus, in practice, the majority of studies have been atheoretical and to date, little information exists which allows to confirm or reject the mediating mechanisms of children’s and adolescents’ physical activity behaviour proposed by the respective models.

### Lacking consideration of moderating variables

In previous studies (e.g., HealthyPEP; 10), physical activity programmes affected boys and girls differently. Therefore, it must be taken into account that intervention effects may not be equally effective across subgroups. Rather it is important to be aware that certain preconditions might have a major influence on the effectiveness of an intervention. Thus, exploring moderating variables to determine for “whom” working mechanisms of interventions are effective is needed [10].

### Lack of intervention content information

The Consolidated Standards of Reporting Trials (CONSORT) statement for randomised trials emphasises the need to provide details of the delivery and description of the different components of the interventions [11]. Nevertheless, in most studies detailed information about the intervention content and the behaviour change techniques employed, which refer to the observable component of an intervention designed to alter causal processes that regulate behaviour, lacks [12]. Hence, a clear link between the intervention components and the changes in physical activity behaviour cannot be made [9].

## Theoretical framework

**Fig. 1:**
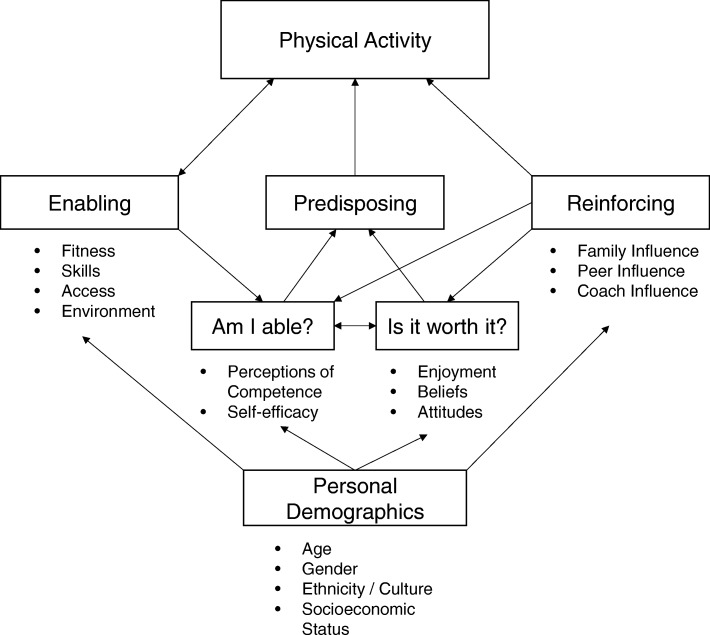
Youth Physical Activity Promotion Model (adapted from Welk, 1999)

In this study, the Youth Physical Activity Promotion Model (YPAPM; 14) is taken as a basic framework (Fig. 1). Based on a social-ecological model approach [13], it suggests that the combination of both individual-level and environmental/policy-level factors can directly and indirectly influence physical activity behaviour.

The YPAPM provides a broad overview of factors associated with physical activity behaviour in children and adolescents and includes constructs that are in turn essential factors in other models. By including the constructs *perceived competence* and the social *influence of family, peers* and *teachers* the YPAPM reflects large parts of the self-determination theory [14]. According to this theory, the level of satisfaction of the basic psychological needs *competence*, *relatedness* and *autonomy* decides whether a person is *intrinsically motivated* to engage in a certain behaviour or not. Mostly cross-sectional studies have confirmed the positive influence of *intrinsic motivation* on physical activity. It is less clear though if positive changes in *intrinsic motivation* – and more precisely in the three underlying basic psychological needs – can serve as significant mediators in interventions trying to promote physical activity of children and adolescents [15]. However, there are a few longitudinal studies pointing to the promotion of the basic psychological needs as a promising means for increasing physical activity in youth. Standage, Gillison [16] showed that in a sample of secondary school students, autonomy support by the physical education (PE) teacher led to a higher satisfaction of the basic psychological needs in this specific context. The subsequently higher intrinsic motivation to participate in PE made the students participate in leisure time sports more autonomously which in turn predicted the objectively measured leisure time physical activity. In a study by McDavid, Cox [17] examining sixth graders over a period of three years, students’ satisfaction of autonomy and relatedness as well as their intrinsic motivation in the context of PE significantly predicted the development of leisure time physical activity. Finally, in a study by Chatzisarantis and Hagger [18] autonomous forms of motivation served as mediators of the effect that the promotion of the basic psychological needs by PE teachers had on adolescent students’ physical activity.

Another construct included in the YPAPM, *self-efficacy,* is the key factor in Bandura’s social cognitive theory [19]. In general, perceived self-efficacy refers to beliefs in one’s capabilities to organize and execute the courses of action required to produce given levels of attainments [19]. To date, Lubans, Foster [8] and van Stralen, Yildirim [9] have conducted two systematic reviews consisting of seven and 18 studies, respectively, that analyse physical activity intervention studies, in which a mediation analysis was carried out. The most promising mediator has been *physical activity self-efficacy*, which explicitly refers to the strength of the belief in one’s own ability to be physically active.

The social support of family and peers is another relevant factor concerning the physical activity of children and adolescents. Together with the aforementioned constructs it completes the reinforcing components for physical activity in the YPAPM. While peers seem to play an important role throughout childhood and adolescence [20], the influence of the parental support affects children’s physical activity especially until the age of 15 [21].

In short, the constructs of *intrinsic motivation*, which in turn depends on the levels of the three basic needs *autonomy*, *competence* and *relatedness* [22], as well as the constructs of *self-efficacy* [19] and *social support* hold a solid theoretical and empirical (e.g., [23, 24]) basis concerning their relevance for physical activity. The intervention programme to be presented here primarily concentrates on the promotion of the three basic needs. Concerning the conceptual similarities of competence and self-efficacy as well as relatedness and social support, the students’ self-efficacy and social support are assessed, too. Therefore, when evaluating the effects of the intervention programme it will be examined for each of these constructs if and to what extent it mediates the desired intervention effect on physical activity.

## Aims of the study

### Does the intervention programme increase the average girls’ physical activity level?

The first objective of this project is to implement and evaluate an intervention programme designed to promote 6th grade girls’ physical activity levels both during PE and outside of school. Offering modified PE classes to promote girls’ physical activity levels in PE and outside of school will be the centrepiece of the intervention. In order to reach 60 min of moderate-to-vigorous physical activity per day, recommended by the WHO [1], physical activity must take place on several occasions during the course of the day. Therefore, the intervention programme is designed to promote girls’ physical activity not only during PE lessons but also during their free time in the afternoon. PE teachers will be trained to carry out the intervention programme. This training will include guidelines and specific materials on how the prepared lesson plans should be put into practice in order to address the constructs of *autonomy*, *competence* and *relatedness,* which are in turn deemed important in terms of increasing physical activity.

*Hypothesis 1*: The intervention programme will significantly increase girls’ physical activity.

### What are the mechanisms of physical activity behaviour change in 6th grade girls?

**Fig. 2:**
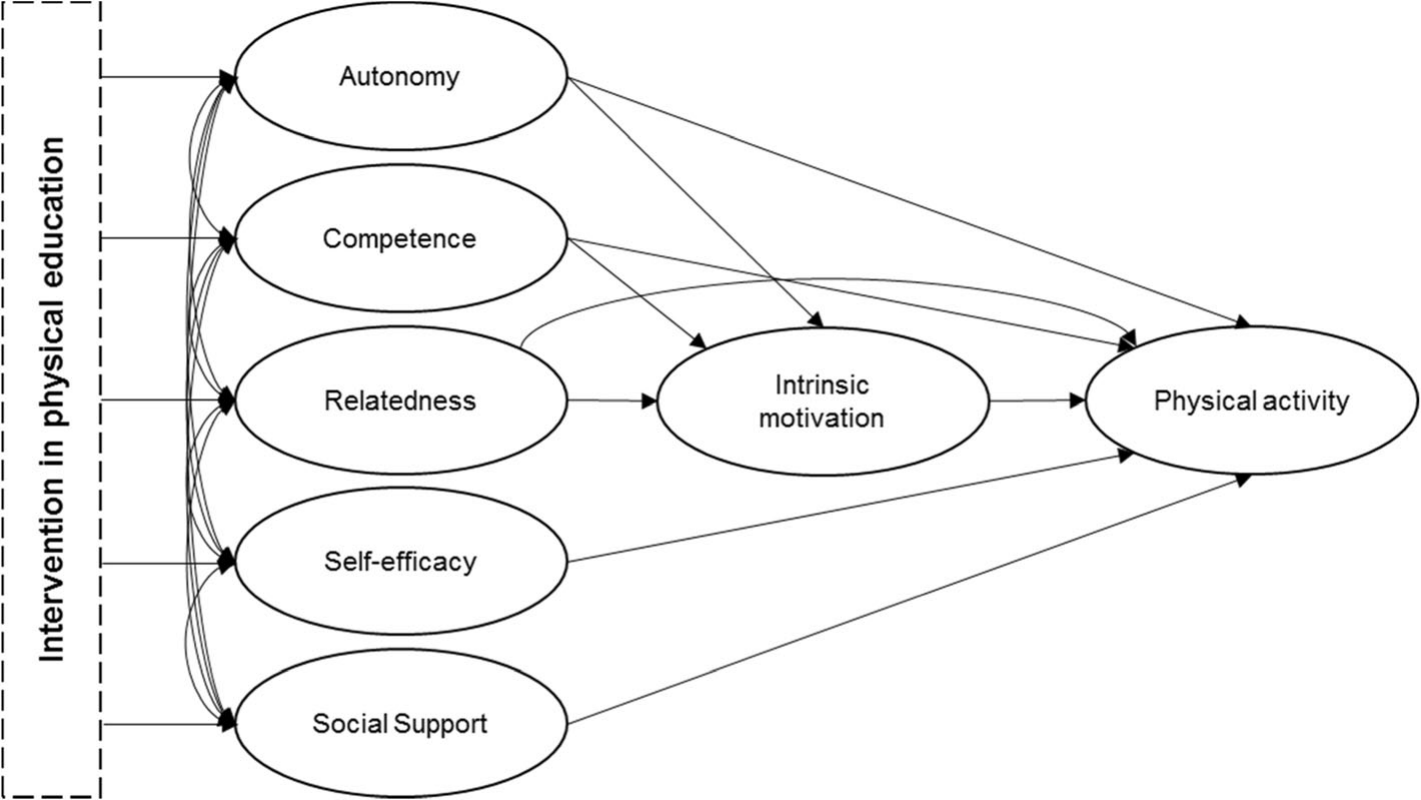
Model of the mediating mechanisms of physical activity to be examined in this project

Based on the YPAPM [25] and more specifically, the self-determination theory [22] and the social cognitive theory [19], it will be examined if positive changes in the basic psychological needs *autonomy*, *competence* and *relatedness* as well as in the adjacent constructs of *self-efficacy* and *social support* lead to an increase in *intrinsic motivation* and thereby cause the desired increase in physical activity (see Fig. 2). Additionally, it will be tested if the three basic psychological needs each have a direct influence on physical activity. Moreover, it will be examined if positive changes in *self-efficacy* and *social support* lead to an increase in physical activity in addition to the increase produced by *intrinsic motivation* and the underlying basic psychological needs.

To this end, several consecutive hypotheses are made.

*Hypothesis 2a*: By following the elaborated lesson plans, the trained teachers will be able to *support girls’ autonomy, competence* and *relatedness* during PE.

*Hypothesis 2b*: The teachers’ support of these potential mediators will result in higher *satisfactions in girls’ autonomy, competence* and *relatedness* in the context of PE.

*Hypothesis 2c*: This will give rise to higher satisfactions of the respective constructs as well as of the constructs *self-efficacy* and *social support* in the leisure time context.

*Hypothesis 2d*: The higher levels of satisfaction of the three basic psychological needs will lead to higher levels in the girls’ intrinsic motivation towards physical activity.

*Hypothesis 2e*: Changes in at least one of the constructs *autonomy*, *competence*, *relatedness*, *self-efficacy*, *social support* and *intrinsic motivation* in the leisure time context significantly mediate the positive effect of the intervention programme on the girls’ physical activity levels.

### Do socioeconomic status, BMI, environment and/or teacher moderate the intervention effect on physical activity?

Based on the theoretical framework of the YPAPM, it will be examined whether socioeconomic status, BMI, environmental factors and/or the girls’ impression of their PE teacher have a moderating effect on the intervention effect.

*Hypothesis 3*: Socioeconomic status, BMI, environment and/or teacher characteristics influence the effect of the intervention programme on the girls’ physical activity.

## Methods/design

### Study design

The study design adheres to the CONSORT guidelines [11]. The effects of the intervention programme will be examined in a two-arm cluster randomised controlled trial with one intervention group and one waiting control group. The intervention programme is carried out during the regular PE lessons by the respective teachers. The *control group* receives regular PE lessons. After follow-up data assessment, the control group receives the intervention programme. The intervention programme is carried out for five months followed by a three-month follow-up period within one school year.

**Fig. 3:**
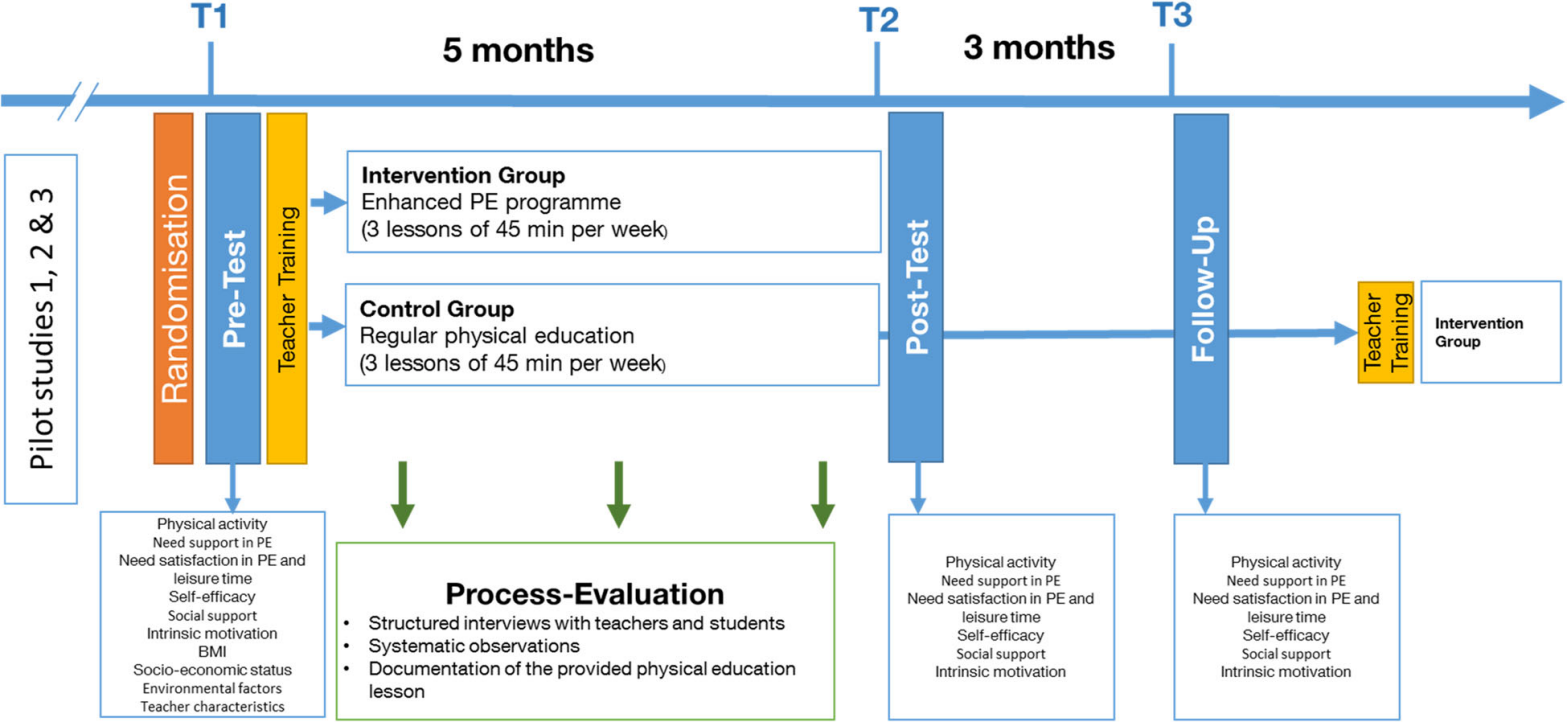
Study design

The majority of variables (physical activity; support and satisfaction of autonomy, competence, relatedness in context of PE and leisure time; intrinsic motivation; self-efficacy; social support) is assessed at three time points: baseline (T1), post-intervention (T2) and follow-up (T3) (see Fig. 3). Socioeconomic status, BMI, teacher characteristics and environmental factors are measured only at baseline. Additionally, in order to monitor to what extent the intervention programme is actually implemented by the respective teachers during the main study, a process evaluation will be carried out. (see Additional file 1: Table S1).

### Study sample

The study sample consists of 6th grade girls (11–13 years of age) from lower secondary schools in and around the city of Munich, Germany. This study sample has been chosen for the following reasons:

- Behaviour patterns are less definite in young adolescents than in adults and can be more easily altered [26]. Additionally, compared to children, young adolescents have the capability to make decisions about their behaviour on a higher cognitive level, which is a prerequisite for the intervention programme to work.
- The results of previous projects indicate differing effects of a school-based health promotion physical activity programme on girls and boys [27, 28]. Therefore, in this study we focus on girls, who are the population most in need for physical activity promotion programmes due to their low physical activity levels [4]. Additionally, in Bavaria PE classes normally are separated by gender and therefore, from a practical perspective it is hardly possible to consider boys and girls in the same classes at the same time.
- The setting of lower secondary schools has been chosen because here, the percentage of students from lower socio-economic status groups is higher compared to the other secondary schools in Germany [29]. The physical activity levels of children and adolescents, and especially girls from a low socioeconomic background, are particularly low [30, 31].

### Sample size calculation

The sample size needed to detect the intended increase in physical activity from baseline to post-intervention was calculated using the formula by Rutterford, Copas [32], who consider several aspects of cluster randomisation, e.g., the intended effect of the intervention, the estimated intracluster correlation (ICC), potentially variable cluster sizes and the implied levels of significance and power. To include appropriate figures for the respective parameters, it is referred to previous research. An intervention-based increase of twenty minutes per day of moderate-to-vigorous physical activity is estimated based on the systematic review by Camacho-Minano, LaVoi [33]. An ICC at the class-level of 0.1 appears appropriate based on the study by Lonsdale, Rosenkranz [34]. Additionally, to account for variable cluster sizes (i.e. number of students within the respective classes) in the present study, we estimate a variation coefficient of the cluster size of 0.25. By inserting the specified figures for the respective parameters and setting an alpha of 0.05 and a power level of 80%, a sample size of at least 467 is required to detect the intended baseline-intervention change in physical activity. Finally, taking dropouts into consideration a sample size of 600 is sought.

### Randomisation

Randomisation of the study sample in intervention group and control group takes place on the school level and is done before pre-test data assessment by a researcher of the study team (see Fig. 3). A computer-based random number producing algorithm is generated and completed by an independent researcher to ensure an equal chance of allocation of a school to either group. No matching is undertaken. Factors differentiating between classes or schools are compensated by use of a multilevel analysis (see statistical analysis).

### Procedure

Before the beginning of the main intervention study, *three pilot studies* were carried out to examine 1) the measurement applicability and the quality criteria of the instruments, 2) the feasibility of the teacher training programme and 3) the feasibility of the intervention programme (see Fig. 3).

In *pilot study 1*, 161 sixth grade students filled out questionnaires and wore accelerometers measuring their physical activity for six days. The instruments (see Additional file 1: Table S1) used to assess the constructs of need satisfaction in PE and leisure time, intrinsic motivation, self-efficacy and social support of family and friends had been translated and adjusted to the stage of development regarding language of sixth-graders before. The adapted scales have shown acceptable to good quality criteria.

In *pilot study 2*, six teachers were trained individually by one researcher in one session with two hours at school or university location. The training was carried out with a prepared project portfolio, presentations, interaction forms and visual materials (see Table 1). Following the training, a guided interview was conducted by a second researcher to obtain information about the success of the training regarding a) teaching and mediation competence of the trainer, used methods and materials, consideration and satisfaction of the teachers’ needs and expectations, b) whether the teachers felt in a position to implement the intervention as planned, areas in which they did not feel sufficiently competent or anticipate possible challenges or difficulties and c) to receive teachers’ comments on improvements in the teacher training and general remarks.

**Table 1:** Teacher training programme

	Content	Detailed description
Introduction	• *Welcome and programme overview*	In the introductory section, a general overview of the project is given after the welcome part. Subsequently, the underlying theories and current state of research are comprehensively addressed. The focus will be on an understanding of the promoted constructs within the intervention programme and of the corresponding behaviour change techniques.
	• *Project introduction*	
	• *Theoretical background*	
	o Current state of research: theories (YPAPM, self-determination theory, social cognitive theory)	
	o Constructs: autonomy, competence, relatedness, self-efficacy, social support	
	o Behaviour Change Techniques	
Main part	• *Intervention programme*	On the basis of a detailed discussion of the lesson plans, the teacher should develop a feeling for how to teach the PE lessons so that the constructs can be promoted. Remarks and challenges that may arise during the implementation of the lessons are addressed and discussed. The main part ends with a discussion of the study organisation, especially the offering of continuous mentoring throughout the intervention period.
	o Structure of the project portfolio	
	o (series of teaching lessons, additional materials)	
	o Series of lesson plans	
	­ Structure and overview	
	- Teaching units and curriculum	
	- Focus on single lesson plans and the applied behaviour change techniques	
	- General remarks and possible challenges	
	• *Study organisation*	
	o Timeline	
	o Methods of data collection	
	o Mentoring (phone, e-mail)	
End	• *Discussion and closing-remarks*	Questions are clarified, further suggestions are made and a conclusion is carried out.

In *pilot study 3*, each of the six trained teachers carried out five PE lessons of one teaching unit (fitness & health, dance, gymnastics, basketball, soccer) and provided information on a) the feasibility of the lessons according to different criteria (content, goals, time, etc.) using a checklist provided by the researchers and b) the overall lesson plans based on notes the teachers took during the lesson. Additionally, using a modified version of the System for Observing Fitness Instruction Time (SOFIT), the extent to which the central constructs of the intervention programme were promoted within the PE lessons was captured. Therefore, the measuring instrument was adapted in such a way that the original three observation categories “physical activity”, “lesson content/context” and “teacher behaviour” were replaced by the promotion of the three constructs “autonomy”, “competence” and “relatedness”. In addition, the observation interval was extended from ten to 30 s. This systematic observation was carried out in two of the five PE lessons held by each teacher.

In order to recruit a study sample of 600 girls several study waves will be necessary. The first study wave was conducted in the school year 2017/2018 and a second study wave 2018/2019 is currently under way.

### Intervention programme

The intervention programme is delivered by the PE or regular class teachers during PE classes. The teachers carrying out the intervention programme are provided with all the materials required for the implementation of the intervention programme during the teacher training session before the programme begins.

The materials include examples of how different behaviour change techniques can be applied to address the girls’ *autonomy*, *competence* and *relatedness* during PE. Most importantly, they receive fully elaborated lesson plans. These lessons combine the requirements of the PE curriculum and the behaviour change techniques by Michie, Richardson [12] in order to establish a change in the targeted mediating variables (see Table 2). The teachers will be asked to implement the lesson plans as thoroughly as possible during the five-month intervention period. Due to the extensive conceptual similarities of self-efficacy and competence as well as relatedness and social support, it is refrained from presenting special behaviour change techniques for the constructs of self-efficacy and social support.

**Table 2:** Theory-based mediators and corresponding behaviour change techniques

YPAPM	Theory	Mediator	Behaviour change techniques (13)
Predisposing Individual level	Self-determination theory	Competence	(2.2) Feedback on behaviour: The teacher provides feedback on performance of the physical activity behaviour during PE and informs the students whether they were active during the lesson and how they can improve their activity levels. (8.7) Graded tasks: The teacher sets easy-to-perform tasks in and after PE, making them increasingly difficult, but achievable, until behaviour is performed. (8.1) Behavioural practice/rehearsal: Sufficient practice time should be included in each PE lesson. Moreover, practice or rehearsal of the performance of the behaviour should be prompted once or more times in a context or at a time when the performance may not be necessary, in order to increase habit and skill without any social pressure.
	Self-determination theory	Autonomy	Providing choice [38]: The teacher supports students’ autonomy during PE by providing them a choice between different contents during the PE lesson and for different activities during the afternoon.
Reinforcing Interpersonal	Self-determination theory	Relatedness	(3.1) Social support: Teachers advise on, arrange or provide social support (e.g. from friends and parents) or non-contingent praise or reward for performance of the behaviour.

### Measures

All measures used in this study can be found in the Additional file 1: Table S1.

### Statistical analysis

Structural equation modeling in R will be used to examine the scientific questions outlined above. To evaluate the effect of the intervention, a multiple group analysis with the intervention and control group will be performed. To account for the clustered nature of the study sample, physical activity will be included in four levels. Level 1 will be the repeated measurements for each individual, level 2 will be the individual itself in which the repeated measurements are nested. Level 3 stands for the class the individual is part of. The class is again nested within a certain school, which represents level 4. The basis for this approach is the assumption, that physical activity is not only influenced by the individual’s personal attitudes and preferences but also by the attitudes and behaviour of the class teacher as well as by the school environment. In a further step, the model will be explicitly extended by the potential moderators BMI, socioeconomic status as well as characteristics of teacher and environment to check if they exert a significant influence on the effect of the intervention.

To gain a deeper insight into the mechanisms of physical activity behaviour change, the girls’ physical activity will be regressed onto the potential mediators. This way, it can be examined which mediators are of incremental importance in relation to the other mediators included in the model concerning the promotion of physical activity. Thereby, the assumed mediators will each be included in three levels.

### Data handling, storage, and monitoring

All data is stored on central servers of the Technical University of Munich. These servers, administered by the Leibniz-Rechenzentrum, meet the high standards for data safety in Germany. Access to personal data will be restricted to the research team. Names and participant details will not be passed onto any third parties and no named individuals will be included in the write up of the results. In order to ensure efficient reuse of the data, other researchers will be given access to the research data through a secured password (provided on request by the principal investigator). The collected and generated data will be archived for at least 10 years.

## Discussion

Physical inactivity is deemed to be the fourth leading cause for premature death [35]. Only a minority of children and adolescents in Germany fulfil the guideline of the WHO of at least 60 min of moderate-to-vigorous physical activity per day [36]. Children and adolescents with a lower socioeconomic background and especially girls are regarded as a high-risk group in terms of physical inactivity [37]. Therefore, theory-based intervention programmes promoting physical activity are needed.

Aim of this study is to examine the effects of CReActivity, an intervention programme based on the self-determination theory and the social cognitive theory, on girls’ physical activity both during PE lessons and in leisure time. Additionally, the extent to which autonomy, competence, relatedness, social support and self-efficacy in girls mediate the effect of the intervention programme on their physical activity will be examined. Moreover, the potential moderating role of socioeconomic status, environmental factors, teacher characteristics and BMI will be tested.

Beyond the examination of these explicit hypotheses stated in the manuscript, the findings from this project can be used in a more general sense. The importance of physical activity promotion both in and out of school through PE is grounded in the curriculum. The didactical concepts and PE teaching methods with respect to the aim of leading an active life can be improved. Modifications in the education of future schoolteachers during their university studies can be established, and, simultaneously, further education teaching modules can be developed for current teachers based on the study findings.

## Additional file

Additional file 1: **Table S1.** Measurement instruments (DOCX 19 kb)

## Abbreviations

BMI: Body mass index
CONSORT: Consolidated Standards of Reporting Trials
HBSC: Health Behaviour in School-aged Children
ICC: Intracluster correlation
PE: Physical education
SOFIT: System for Observing Fitness Instruction Time
WHO: World Health Organization
YPAPM: Youth Physical Activity Promotion Model
