# Supplementary material for: A school-based intervention based on self-determination theory to promote girls' physical activity: study protocol of the CReActivity cluster randomised controlled trial
Source: BMC Public Health. 2019 May 6;19:519. doi: 10.1186/s12889-019-6817-y (PMC6503350; doi:10.1186/s12889-019-6817-y)
Supplement: Supplementary file 1 — Table S1. Measurement instruments (DOCX 19 kb) [file 12889_2019_6817_MOESM1_ESM.docx]

Additional table 1: Measurement instruments

|  | **Variables** | **Instrument** | **Description** |
| --- | --- | --- | --- |
| **Primary outcome** | Physical activity (overall) | Accelerometers  ActiGraph wGT3X-BT | Girls wear the accelerometers placed on their waists with an elastic waistband for seven consecutive days. They are asked to wear the monitor whenever awake, except during water-based activities. |
|  | Physical activity (during PE) | System for Observing Fitness Instruction Time (SOFIT)  (36) | The SOFIT tool assesses data on student activity levels during PE (MVPA (moderate-to-vigorous physical activity); VPA (vigorous physical activity); lying down, sitting, standing, and walking), the lesson context, and teacher behaviour. |
| ***Mediators*** | Need satisfaction:  Autonomy  Competence  Relatedness | Basic Psychological Needs Scale (BPNS)  (37) | The Basic Psychological Needs Scale (Chen et al., 2015) measures the general satisfaction and frustration of the three constructs autonomy, competence and relatedness with 24 items on a 5-point Likert scale. The 12 satisfaction items were translated, adapted to the context of physical activity and pilot tested. |
|  | Self-efficacy | Social-cognitive measures (38) | Self-efficacy is measured using an eight-item questionnaire validated with a sample of sixth and eighth grade girls. A German version was pilot tested with sixth grade students in Germany. A 5-point Likert-type response format is used. |
|  | Social support | Social cognitive measures (38) | Social support by family (4 items) and friends (3 items) is measured with two subscales validated with a sample of sixth and eighth grade girls. A 5-point Likert-type response format is used. After translating the items into German they were pilot tested with sixth grade students. |
|  | Behavioural regulation in exercise  Intrinsic motivation | Behavioural Regulation in Exercise Questionnaire-2 (BREQ-2) (39) | BREQ-2 assesses the behavioural regulation in exercise with five subscales: intrinsic motivation (4 items), amotivation (4 items), external regulation (4 items), introjected regulation (3 items) and identified regulation (4 items). The scales were translated and pilot tested with sixth grade students in Germany. Responses are provided on a 5-point Likert-type scale. |
| ***Moderators*** | Socio-economic status | International Socio-Economic Index of Occupational Status-08 (ISEI-08) (40) | The girls are asked about the occupation of their parents. Afterwards, each child is allocated an ISEI value between 16 and 90 (40), based on the International Standard Classification of Occupations (ISCO). The value of the parent who is rated higher is the relevant one. |
|  | BMI | - Weight scale - Stadiometer | Measurement of height and weight |
|  | Environmental factors | ALPHA short (41) | Environmental factors of relevance for physical activity are assessed with 10 items. |
|  | Teacher characteristics | Items from SPRINT study (42) | The girls’ impression of their PE teacher is assessed with six items taken from the SPRINT study (42). Girls are asked to fill out a semantic differential rating their teacher as strict or indulgent, uncomprehending or understanding, unprepared or well prepared, unathletic or athletic, unjust or just and unhumorous or humorous. |
| ***Process evaluation*** | Dose delivered, received & context | - Interviews with teachers and students - Systematic observation of PE - Documentation of the lessons carried out | - Every teacher of the intervention group and two students from each class are interviewed with standardised interview questionnaires to gain information about the extent to which the intervention was delivered, received and about the context in which it was carried out. - 3 Systematic observations in each class during PE are carried out to examine whether the central concepts of autonomy, competence and relatedness were addressed by the teacher. - The teachers will document which of the provided PE lessons they have carried out. |
|  | Need support:  Autonomy  Competence  Relatedness | Adolescent Psychological Need Support in Exercise Questionnaire  (43) | PE teacher’s support of the three basic needs autonomy, competence and relatedness is measured by three items for each need. |
